# A unified and simple medium for growing model methanogens

**DOI:** 10.3389/fmicb.2022.1046260

**Published:** 2023-01-10

**Authors:** Daniel Dzofou Ngoumelah, Falk Harnisch, Snorre Sulheim, Tonje Marita Bjerkan Heggeset, Ingvild Haugnes Aune, Alexander Wentzel, Jörg Kretzschmar

**Affiliations:** ^1^Biochemical Conversion Department, DBFZ Deutsches Biomasseforschungszentrum gemeinnützige GmbH, Leipzig, Germany; ^2^Department of Environmental Microbiology, Helmholtz Centre for Environmental Research - UFZ, Leipzig, Germany; ^3^Department of Biotechnology and Nanomedicine, SINTEF Industry, Trondheim, Norway

**Keywords:** methanogenic archaea, medium adaptation, microbial electrochemical technology, bioelectrochemical systems, doubling time, specific growth rate

## Abstract

Apart from their archetypic use in anaerobic digestion (AD) methanogenic archaea are targeted for a wide range of applications. Using different methanogenic archaea for one specific application requires the optimization of culture media to enable the growth of different strains under identical environmental conditions, e.g., in microbial electrochemical technologies (MET) for (bio)electromethanation. Here we present a new culture medium (BFS01) adapted from the DSM-120 medium by omitting resazurin, yeast extract, casitone, and using a low salt concentration, that was optimized for *Methanosarcina barkeri*, *Methanobacterium formicicum,* and *Methanothrix soehngenii*. The aim was to provide a medium for follow-up co-culture studies using specific methanogens and *Geobacter* spp. dominated biofilm anodes. All three methanogens showed growth and activity in the BFS01 medium. This was demonstrated by estimating the specific growth rates ( μ ) and doubling times ( $t_{d}$ ) of each methanogen. The μ and $t_{d}$ based on methane accumulation in the headspace showed values consistent with literature values for *M. barkeri* and *M. soehngenii*. However, μ and $t_{d}$ based on methane accumulation in the headspace differed from literature data for *M. formicicum* but still allowed sufficient growth. The lowered salt concentration and the omission of chemically complex organic components in the medium may have led to the observed deviation from μ and $t_{d}$ for *M. formicicum* as well as the changed morphology. 16S rRNA gene-based amplicon sequencing and whole genome nanopore sequencing further confirmed purity and species identity.

## Highlights

- Methanogens with different metabolisms can be cultured in the same growth medium.
- Primers for metabarcoding of the studied archaea were designed and verified.

## 1. Introduction

Methanogenic archaea represent a highly diverse group of microorganisms performing methanogenesis, that is producing methane (CH_4_) under strict anoxic conditions (Dworkin et al., 2006; Buan, 2018). They obtain energy by metabolizing hydrogen and carbon dioxide, acetate, formate, or short-chain methylated compounds like methanol (Neil and James, 1980; Bryant and Boone, 1987a; Long et al., 2017). Depending on the taxon and the respective metabolism, methanogenesis involves either the reduction of CO_2_ with H_2_ or the disproportionation of small organic molecules, such as acetate, formate, methanol and methylamine to methane and CO_2_ (Dworkin et al., 2006; Bar-Even et al., 2012; Goyal et al., 2016; Kouzuma et al., 2017). In anaerobic digestion (AD), especially hydrogenotrophic and some mixotrophic methanogens consume H_2_ that is produced during acido- and aceto-genesis, hence providing a natural hydrogen sink that facilitates anaerobic degradation of organic compounds to acetate and other volatile fatty acids (VFA; Kouzuma et al., 2015; Camacho and Ruggeri, 2018). The reduction of CO_2_ to CH_4_ during methanogenesis can take place either (1) *via* mediated interspecies electron transfer (MIET), in which methanogens use endogenous (H_2_) or exogenous mediators (redox-active compounds such as neutral red) as electron carriers (Park et al., 1999; Rotaru et al., 2014b; Mayer et al., 2019), or (2) direct interspecies electron transfer (DIET), in which, e.g., members of the *Methanosarcinaceae* and *Methanosaetaceae*, establish physical cell-to-cell contact with electroactive bacteria (EAB) to directly receive electrons released by the latter during oxidation of mainly acetate (Rotaru et al., 2014a; Yee and Rotaru, 2020; Yin et al., 2020).

The industrial application of methanogens within the AD process gained momentum in the mid-twentieth century. Since then, AD has been used to valorize the energy contained in organic waste and transform it into biogas which is used as a renewable energy carrier (Camacho and Ruggeri, 2018). The increasing industrial application of methanogenesis has led to growing efforts to increase the efficiency and sustainability of AD. Here, microbial electrochemical technologies (MET) are promising, e.g., as microbial fuel cells to treat effluents from biogas production, provide alternative pathways for CH_4_ production using microbial electrochemical methanation (Deutzmann and Spormann, 2017; Mayer et al., 2019; Kracke et al., 2020), or for developing microbial electrochemical sensors for online monitoring of AD (Hill et al., 2020). The combination of MET and AD requires the examination of specific interactions between methanogens and EAB, such as DIET, and their consequences for electroactive biofilms in AD environments. One specific example is the application of microbial electrochemical sensors using *Geobacter* spp. dominated biofilms on graphite electrodes for AD process monitoring (Kretzschmar et al., 2018).

Examination of interactions between microorganisms from different domains, kingdoms, phyla etc. in the same environment or at identical conditions requires compromises when adapting growth conditions like, e.g., pH, temperature, gas atmosphere, salinity, trace elements and vitamins, and redox potential. Hence, growth media and setups that enable the implementation of both, EAB including electrodes and different kinds of methanogenic archaea, are highly demanded. The growth medium is of specific interest as the existing recipes for different methanogens differ substantially and contain ingredients that can interfere with EAB or electrodes. These chemicals can be redox-active compounds (e.g., the redox-sensitive dye resazurin, used to monitor redox potential during methanogen cultivation) that can interfere with the electrodes in the electrochemical setup (Lian et al., 2016). For instance, the biochemical standard potential, $E^{0^{\prime }}$ of the resofurin/di-hydroresofurin complex at pH 7 is −51 mV vs. the standard hydrogen electrode (SHE) that is well within the usually applied range of electrode potentials in microbial electrochemical systems. Additionally, some compounds can be used as terminal electron acceptors by EAB, such as elemental sulfur or iron-(III)-salts (Sun et al., 2014; Yang et al., 2015). Similarly, although casitone (peptone from casein) and yeast extract have been recognized as significant factors or stimulators during methanogen growth (Dworkin et al., 2006), they could interfere with EAB when combining methanogens and MET, e.g., in the case of yeast extract being used as a mediator by EAB (Sayed et al., 2015). Certain model methanogens, like, e.g., *Methanohalophilus zhilinae,* require (extreme) halophilic or halotolerant growth conditions (Enzmann et al., 2018). However, high ionic strength can affect the performance of EAB (e.g., *Geobacter* spp. biofilm anodes) as their salt tolerance varies widely (Lefebvre et al., 2012; Liu et al., 2014; Sun et al., 2014; Kretzschmar et al., 2018). Therefore, combining methanogens and EAB in MET for further studies and especially for practical applications will require tailoring of the salt concentrations, enabling well controlled cultivation of methanogens and EAB. Finally, a well-defined medium will greatly facilitate future analytical (e.g., exometabolome) assays or modeling efforts (Bernstein et al., 2021).

Currently, the cultivation of specific methanogenic strains is associated with specific growth media, hampering their applications, e.g., for studies that involve methanogens and EAB. Such studies are highly interesting as AD-MET combinations are expected to increase the overall efficiency of AD and therefore, could contribute to maximize the energetic valorization of organic residues (De Vrieze et al., 2018). Furthermore, these growth media are usually complex and require many supplements. Therefore, it is of utmost importance to simplify, unify, and adapt media for specific methanogens of interest or design new general media resembling those of other model microorganisms, e.g., EAB, in which a wide range of methanogens could thrive. To the best of our knowledge, the design of more general methanogenic media is still poorly explored. In the present study, we designed and used the BFS01 medium, adapted from the DSM-120 medium, to grow *Methanosarcina barkeri* (mixotrophic methanogen), *Methanobacterium formicicum* (hydrogenotrophic methanogen), and *Methanothrix soehngenii* (acetotrophic methanogen). Further, we investigated whether and how the use of a low salt concentration and the omission of certain, partially redox active compounds (e.g., resazurin, yeast extract, casitone) in the BFS01 medium affected the morphological and physiological parameters of each methanogen. In particular, the cell shape and standard growth parameters i.e., specific growth rate ( μ ) and doubling time ( $t_{d}$ ) of each methanogen cultured in the BFS01 medium were assessed. To verify species identity and absence of contaminants, each methanogen was analyzed at the DNA level using whole genome nanopore sequencing and 16S rRNA gene-based amplicon sequencing.

## 2. Materials and methods

All microbiological experiments were conducted under strictly axenic and anoxic conditions. All chemicals were of analytical or biochemical grade. Experiments were performed as independent biological triplicates (*n* = 3).

### 2.1. Methanogenic strains

*Methanobacterium formicicum* MF (DSM 1535) and *Methanosarcina barkeri* MS (DSM 800) strains, previously purchased from the German Collection of Microorganisms and Cell cultures (DSMZ), were provided by the working group microbiology of anaerobic systems at the department of Environmental Microbiology of the Helmholtz Centre for Environmental Research, Leipzig, Germany. *Methanothrix soehngenii* GP6 (DSM 3671) strain was provided by the microbiology laboratory at the department for agrotechnology and food sciences of Wageningen University and Research, Wageningen, The Netherlands.

### 2.2. Setup preparation

The experimental setup for culturing each methanogenic strain consisted of 200 ml serum bottles closed with butyl rubber stoppers and aluminum crimp seals (LABSOLUTE, Th. Geyer GmbH, Germany). The aluminum crimp seals had a 9 mm opening to allow sampling *via* injection needles. Prior to the experiments, the serum bottles were thoroughly washed and dried. 500 μl of distilled water was added to each bottle, closed with aluminum foil and autoclaved at 121° C and 1.2 bar for 20 min. The bottles were subsequently placed in the anaerobic bench/GloveBox (Coy Laboratory Products Inc) and left open overnight under N_2_:H_2_ (97:3, v/v) atmosphere. After at least 12 h, 500 μl anoxic water prepared according to (Logroño et al., 2020) was added to each serum bottle, closed with previously autoclave-sterilized butyl rubber stoppers and clamped with aluminum caps. All bottles were re-sterilized by autoclaving at 121°C and 1.2 bar for 20 min.

### 2.3. Growth medium

Based on the composition of the recommended or commonly used media for each of the three methanogenic strains (see Supplementary Table S1), the BFS01 medium was designed (see Table 1).

**Table 1:** BFS01 medium used for culturing the methanogenic strains.

Component	Amount	Unit
K_2_HPO_4_	0.35	g L^−1^
KH_2_PO_4_	0.23	g L^−1^
NH_4_Cl	0.50	g L^−1^
MgCl_2_ × 6 H_2_O	0.41	g L^−1^
CaCl_2_ × 2 H_2_O	0.25	g L^−1^
NaCl	2.25	g L^−1^
FeCl_2_ × 4H_2_O	1.42	mg L^−1^
NaHCO_3_	0.85	g L^−1^
C_3_H_8_ClNO_2_S × H_2_O	0.30	g L^−1^
Trace element solution SL-10	1.00	ml L^−1^
Wolin’s vitamin solution-10	1.00	ml L^−1^

The adaptation aimed to omit all redox active components (e.g., resazurin) to avoid electrochemical interferences as well as additional carbon sources (e.g., yeast extract and casitone) in studies that may involve methanogens and EAB. The salt concentration was also adapted to be suitable for the model EAB *Geobacter sulfureducens* and *Geobacter anodireducens*. All compounds (except sodium bicarbonate, vitamin, trace elements, and cysteine) were weighed into a 1 l Duran bottle (Schott AG) and dissolved with distilled water. The mixture was sparged with oxygen-free N_2_ gas for 60 min, tightly sealed with a butyl rubber stopper and a screw cap with 33 mm opening, and sterilized by autoclaving at 121°C and 1.2 bar for 20 min. The other stock solutions (i.e., sodium bicarbonate, vitamins, trace elements, and cysteine) were made anoxic by stirring in the anaerobic bench under N_2_:H_2_ (97:3, v/v) atmosphere for 30 min at 500 rpm. Subsequently, each stock solution was sterilized by filtration into closed, anoxic, sterile 250 ml Duran bottles, using a sterile 0.2 μm filter-needle-syringe arrangement (Sartorius Stedim Biotech GmbH, Germany) previously made anoxic by flushing with sterile oxygen-free N_2_. Stock solutions used as carbon and energy source, i.e., 2 mol L^−1^ sodium acetate and 2 mol L^−1^ sodium formate, as well as solutions used to adjust pH, i.e., 32% sodium hydroxide and 3 mol L^−1^ hydrochloric acid, were prepared in 100 ml Duran bottles, sealed with butyl rubber stoppers and screw caps, flushed with oxygen-free N_2_ for 30 min and sterilized by autoclaving at 121°C and 1.2 bar for 20 min. Because of its low vapor pressure, methanol was directly filter-sterilized and then rendered anoxic by flushing with sterile oxygen-free N_2_ for 10 min. Stock solutions of vitamin and trace elements were stored at 4°C while the other stock solutions were stored at room temperature in the dark. Fully supplemented growth medium for each methanogen was prepared by adding the respective filter-sterilized supplements to the BFS01 medium and their corresponding carbon sources (see Table 2) using a sterile anoxic needle-syringe arrangement. The final pH of each methanogenic growth medium was adjusted as indicated in Table 2 and the prepared media were stored at room temperature in the dark for later use.

**Table 2:** Growth conditions for methanogenic cultures, pH values adjusted according to literature (Neil and James, 1980; Huser et al., 1982; Neil et al., 1982; Touzel et al., 1988).

	Substrate	Gas phase	pH
*Methanosarcina barkeri*	185 mmol L^−1^ methanol, 10 mmol L^−1^ acetate	N_2_:H_2_ ~ (97:3, v/v)	7.1
*Methanobacterium formicicum*	50 mmol L^−1^ formate, H_2_:CO_2_ (50:50, v/v)	H_2_:CO_2_ (50:50, v/v)	7.5
*Methanothrix soehngenii*	40 mmol L^−1^ acetate	N_2_:CO_2_ (50:50, v/v)	7.6

### 2.4. Cultivation of methanogens

Prior to culturing each methanogen in the BFS01 medium, they were routinely precultured in their media recommend by DSMZ (DSM-120 medium for *M. barkeri* and DSM-141 medium for *M. formicicum*, see Supplementary Table S1 for media composition) or CP anaerobic medium (*M. soehngenii*, see Supplementary Table S1 for media composition; Stams et al., 1993), following the recommended procedures. The growth procedure was originally adapted from the Hungate technique (Zeikus, 1977; Balch et al., 1979; Huser et al., 1982) and further modified, as in this study. 127 ml of each fully supplemented, BFS01 medium (see Tables 1, 2) was dispensed into sterile, anoxic serum bottles, using a needle-syringe arrangement.

To avoid significant carryover of yeast extract, trypticase-peptone/casitone and resazurin in the cultures, serum bottles were inoculated separately with 3 ml aliquots of each precultured methanogen corresponding to ~2.3% v/v inoculum and grown in the dark at 37°C until a similar CH_4_ concentration was measured in the headspace of the cultures for several days. The final inoculum was depleted in remaining yeast extract, trypticase-peptone (casitone), and resazurin by culturing twice in the BFS01 medium using ~2.3% v/v of the previous inoculum. Only cultures from the second transfer were used as inocula for subsequent investigations.

Thereafter, 120 ml of fully supplemented BFS01 medium was dispensed into anoxic and sterile serum bottles and inoculated with 10 ml inoculum (corresponding to ~7.7% v/v inoculum) of each methanogen in the late logarithmic phase or early stationary phase, determined by optical density at 600 nm (OD_600_) or CH_4_ concentration in the culture headspaces. After inoculation the *M. barkeri* serum bottles contained 16.50 ± 0.15 mmol L^−1^ acetate, 185 mmol L^−1^ methanol with a 0.01 bar N_2_:H_2_ ~ (97:3, v/v) gas phase, whereas the *M. formicicum* serum bottles contained 55.22 ± 1.42 mmol L^−1^ formate and were flushed for at least 2 min with a sterile H_2_:CO_2_ (50:50, v/v) gas mixture and overpressurized to 1.5 bar. After inoculation, the *M. soehngenii* serum bottles contained 42.17 ± 0.21 mmol L^−1^ acetate with a 0.5 bar N_2_:CO_2_ (50:50, v/v) gas phase. Acetate and formate concentrations of newly inoculated serum bottles were slightly higher than those presented in Table 2 due to the additional carbon input or carryover from the inocula. Uninoculated, fully supplemented BFS01 medium for each methanogen, was set up as sterile control.

All methanogenic cultures were incubated in the dark for 7 weeks at 37°C (New Brunswick Innova® 44 Incubator Shaker Series). *M. barkeri* and *M. formicicum* cultures were maintained under constant orbital shaking at 100 rpm, while *M. soehngenii* cultures were unshaken, the latter being known to have better growth at rest (Huser et al., 1982). During the 7-week culture period, *M. formicicum* cultures were routinely flushed every 3 to 4 days with sterile H_2_:CO_2_ (50:50, v/v) and overpressurized to 1.5 bar, to avoid underpressure due to H_2_:CO_2_ consumption and to remove the produced CH_4_. *M. barkeri* cultures were vented twice a week at 0.01 bar until day 28 to avoid overpressure due to the large amount of CH_4_ produced during methanol feeding (see also equation 5 in Table 3). In contrast, *M. soehngenii* cultures were not vented during the 7-week incubation period, as the pressure in the serum bottles never exceeded the security limit of 1.5 to 2 bar.

**Table 3:** Reactions and different Gibbs free energy changes for methanogenesis at neutral pH (Dworkin et al., 2006).

Energy substrate	Reaction	*ΔG°*′ (kJ/mol of methane)	Eq.
Acetate	$CH_{3}COO^{-}+H_{2}O\to CH_{4}+HCO_{3}^{-}$	−31 kJ mol^−1^	2
Hydrogen	$4H_{2}+CO_{2}\to CH_{4}+2H_{2}O$	−135.6 kJ mol^−1^	3
Formate	$4HCOOH\to CH_{4}+3CO_{2}+2H_{2}O$	−130.1 kJ mol^−1^	4
Methanol	$4CH_{3}OH\to 3CH_{4}+CO_{2}+2H_{2}O$	−104.9 kJ mol^−1^	5
	$CH_{3}OH+H_{2}\to CH_{4}+H_{2}O$	−112.5 kJ mol^−1^	6

### 2.5. Analysis

The growth of each culture was monitored weekly using chemical oxygen demand removal ( ΔCOD ), consumption of acetate and formate, headspace gas composition, OD_600_, cell number, and phase-contrast microscopy to analyze purity and cell morphology.

The COD removal (i.e., the share of organic material that is consumed between two specific time intervals) was measured using COD cuvette tests (LCK 014, Hach-Lange), according to the manufacturer’s instructions.

The COD removal efficiency was calculated as follows:


(1) $$\%COD=\frac{COD_{t_{0}}-COD_{t}}{COD_{t_{0}}}\times 100$$

Where $COD_{t_{0}}$ is the initial COD concentration at *t_o_* and $COD_{t}$ the COD concentration at the sampling point *t* (Nwaigwe and Enweremadu, 2015).

For measuring the acetate and formate concentration in each methanogenic culture over the incubation time, 1 ml aliquots were sampled through the butyl rubber ports right after inoculation and then once a week. The samples were filtered using 0.2 μm syringe filters (Nylon, VWR) and stored at −20°C or analyzed immediately using high performance liquid chromatography (HPLC; Shimadzu Scientific Instruments) equipped with a refractive index detector RID 10A, a prominence diode array detector SPD.M20A, and a HiPlex H column (300 × 7.7 mm, 8 mm pore size, Agilent Technolgies, United States) with a pre-column HiPlex H column (50 mm × 7.7 mm, 8 mm pore size, Agilent Technolgies, United States).

The sample volume for HPLC measurement was 200 μl and the injection volume was 20 μl. 5 mmol L^−1^ sulfuric acid was used as isocratic mobile phase with a flow rate of 0.7 ml min^−1^ at 55°C, over a total run time of 60 min.

CH_4_, H_2_, and CO_2_ concentration in the headspace of each serum bottle were determined weekly during growth. Therefore, before venting or flushing the serum bottles, two replicates of 1 ml gas samples were taken through butyl rubber ports from the headspace of each serum bottle using sterile needle-syringe arrangements rendered anoxic by flushing with sterile oxygen-free N_2_ gas. Each sampled gas was injected into glass vials pre-flushed for 30 min with argon (Argon 4.8, Linde AG). Gas composition was analyzed using a gas chromatograph (GC) equipped with an autosampler (Perkin Elmer Inc., Waltham). The GC was equipped with HayeSep N/Mole Sieve 13X columns and a thermal conductivity detector. The oven and detector temperatures were 60°C and 200°C, respectively. The carrier gas was argon. Each gas sample was analyzed within 4 h after sampling.

The optical density of the liquid phase was determined by photometric measurement at 600 nm (OD_600_) using a spectrophotometer (Genesys 10S UV–Vis, Thermo Scientific). Before sampling for OD_600_ measurements, each bottle was sufficiently hand-shaken. 1 ml of each methanogenic cultures were placed in a cuvette and the OD_600_ was measured.

The purity and cell morphology of each methanogen grown in the BFS01 medium was checked by phase-contrast microscopy using a Zeiss Observer Z.1, equipped with Zen 3.0 software (blue edition), an Axiocam 503 mono Camera, and a plan Apochromat 63x/1,4 Oil Ph3 objective. Before each measurement, the cultures were thoroughly shaken by hand. Subsequently, an aliquot was withdrawn from each culture under strict anoxic and sterile conditions. 2 to 3 drops were placed on a multi-well microscope slide and visualized under the microscope.

The exponential growth phases were estimated by examining the log-values of CH_4_ and OD_600_. Then, by using the method of least squares (Microsoft Excel 2019), the specific growth rate ( μ ) was estimated by fitting a linear curve to the log-scaled values. Additionally, the doubling time ( $t_{d}$ ) based on CH_4_ concentration in the headspace and OD_600_ were determined. More information on the calculation of μ and $t_{d}\;$ of microorganisms is provided in the calculation section of the Supplementary Information (SI), and in the literature (Powell, 1983; Kirchman, 2002).

### 2.6. Molecular biology

Genomic DNA of each methanogen grown in the BFS01 medium was extracted using the Quick-DNA™ Fecal/Soil Microbe Miniprep Kit (ZYMO RESEARCH). DNA concentrations were measured by fluorescence quantification using the Qubit™ dsDNA BR Assay Kit (Thermo Fisher Scientific, cat. Q32852) and a Qubit™ 4 Fluorometer (Thermo Fisher Scientific). The purity of the DNA was evaluated by microvolume absorbance on a DS-11 FX+ (Denovix), where A260/A280 and A260/A230 values around 1.8 and 2.0, respectively, are considered as pure nucleic acid. Amplicon sequencing was used to check the purity of each methanogenic culture. To identify appropriate archaeal primers targeting the V3-V4 region of the 16S rRNA gene typically targeted in metabarcoding studies, 16S rRNA gene sequences were extracted from the published genome assemblies of *M. barkeri* (Genbank accessions: NZ_CP008746, NZ_CP009517, NZ_CP009526, NZ_CP009528, NZ_CP009530), *M. formicicum* (Genbank accessions: NZ_LN734822, NZ_CP006933, LN515531, and BBES01000067 from the BBES01 whole genome shotgun assembly), and *M. soehngenii* (Genbank accession: NC_015416). These 16S rRNA gene sequences as well as the published rRNA gene sequences for strains DSM 800 and DSM 1535 (Genbank accessions: AJ012094 and AF169245, respectively) were then aligned using Clone manager Suite 9.51 (Scientific and Educational Software). The V3-V4 region of the gene was checked for compatibility to the primers Bakt_341F (5′-CCTACGGGNGGCWGCAG-3′, *Escherichia coli* position 341–357) and Bakt_805R (5′-GACTACHVGGGTATCTAATCC-3′, *E. coli* position 805–785; Herlemann et al., 2011; Klindworth et al., 2013). No issues were found for the Bakt_805R primer, while there were two positions in the middle of the Bakt_341F primer that did not match with any of the 3 archaea sequences and would not produce any PCR products (data not shown). A new primer, Arch_F (5′-CCTACGGGGYGCAGCAG-3′) was designed based on the consensus of the archaea in this region. The PCR amplification targeting the V3 and V4 regions of both bacterial and archaeal 16S rRNA genes to detect possible bacteria contamination in the methanogenic cultures was run with a mixture of the degenerated primers F-bacteria (Bakt_341F extended with an Illumina adapter, 5′-TCGTCGGCAGCGTCAGATGTGTATAAGAGACAG-CCTACGGGNGGCWGCAG-3′), F-archaea (Arch_F extended with an Illumina adapter, 5′-TCGTCGGCAGCGTCAGATGTGTATAAGAGACAG-CCTACGGGGYGCAGCAG-3′) and, R-all (Bakt_805R extended with an Illumina adapter, 5′-GTCTCGTGGGCTCGGAGATGTGTATAAGAGACAG-GACTACHVGGGTATCTAATCC-3′) in molar ratios reflecting the number of nucleotide variations found in each primer (i.e., F-bacteria:F-archaea:R-all 3:1:1, v/v). The priming parts of the primers are underlined.

Sequencing libraries were prepared according to the Illumina 16S Metagenomic Sequencing Library Preparation protocol (Illumina Part # 15044223 Rev. B). PCR reaction products were mixed with Gel Loading Dye Purple (6X) B7024S (New England Biolabs) and checked for size and false priming issues by 0.8% agarose gel electrophoresis (SeaKem® LE Agarose, Lonza) in 1x TAE (Thermo Fisher Scientific). The GeneRuler 1 kb DNA Ladder (Thermo Fisher Scientific) was included as a size distribution reference. The amplicon libraries were sequenced on an Illumina MiSeq with the MiSeq Reagent Kit v3 in a paired-end mode and read lengths of 300 bp. Raw sequencing data was demultiplexed and converted to fastq-files in Local Run manager (Illumina). The data were processed using the CLC Genomics Workbench 22.0.1 (Qiagen).

To confirm results from the 16S rRNA gene-based amplicon sequencing and compare to previously published genome sequences from the used strains, whole genome nanopore sequencing was performed. Sequencing libraries were generated using the Ligation Sequencing Kit (Oxford Nanopore Technologies, SQK-LSK109). DNA libraries were loaded onto a Flongle Flow Cell (FLO-FLG001, Oxford Nanopore Technologies) connected to a MinION instrument equipped with a Flongle adapter. The sequencing was controlled by MinKNOW version 21.10.4 (Oxford Nanopore Technologies). The raw nanopore sequence data were base-called using Guppy version 6.0.6 (Oxford Nanopore Technologies) with the dna_r9.4.1_450bps_hac.cfg config file and in high-accuracy mode. Guppy was also used to trim adapter sequences. The base-called reads were filtered based on length (minimum 1,000 bp) and quality (minimum Q10 for *M. barkeri* and *M. soehngenii*, minimum Q7 for *M. formicium* due to the lower sequencing output) using Nanofilt (De Coster et al., 2018). The reads were assembled into contigs with Flye version 2.8.1 (Kolmogorov et al., 2019). The quality of the assemblies was assessed using QUAST version 5.0.2 (Gurevich et al., 2013). Finally, estimated average nucleotide identity (ANI) and alignment percentages (AP) between the assembled genomes and available reference genomes of the three methanogens (downloaded from NCBI, https://www.ncbi.nlm.nih.gov/genome) were calculated by the Whole Genome Alignment Plugin in CLC Genomics workbench v. 22.0 (Qiagen) by employing the Create Whole Genome alignment v. 1.0 and Create Average Nucleotide Identity Comparison 1.0 tools in default setting.

### 2.7. Stoichiometry and Gibbs free energy of relevant methanogenesis pathways

Several reactions are involved in the metabolism of different methanogenic substrates into methane. Table 3 summarizes the different reactions and standard Gibbs free energy for methanogenesis as a function of the carbon (energy) substrate during cultivation of *M. barkeri, M. formicicum*, and *M. soehngenii*.

### 2.8. Statistics

Statistical analysis was performed using Origin Version 2021 (OriginLab Corporation, Northampton, MA, United States, Version 9.8.0.200). Results are presented as mean values with corresponding confidence interval (CI) at 95% confidence level calculated from triplicate analyses (Cumming et al., 2007).

## 3. Results

### 3.1. Growth and morphology of *Methanosarcina barkeri*, *Methanobacterium formicicum*, and *Methanothrix soehngenii* in the BFS01 medium

The growth and morphology of *M. barkeri*, *M. formicicum,* and *M. soehngenii* were monitored using the BFS01 medium for a total period of 7 weeks.

**Figure 1:**
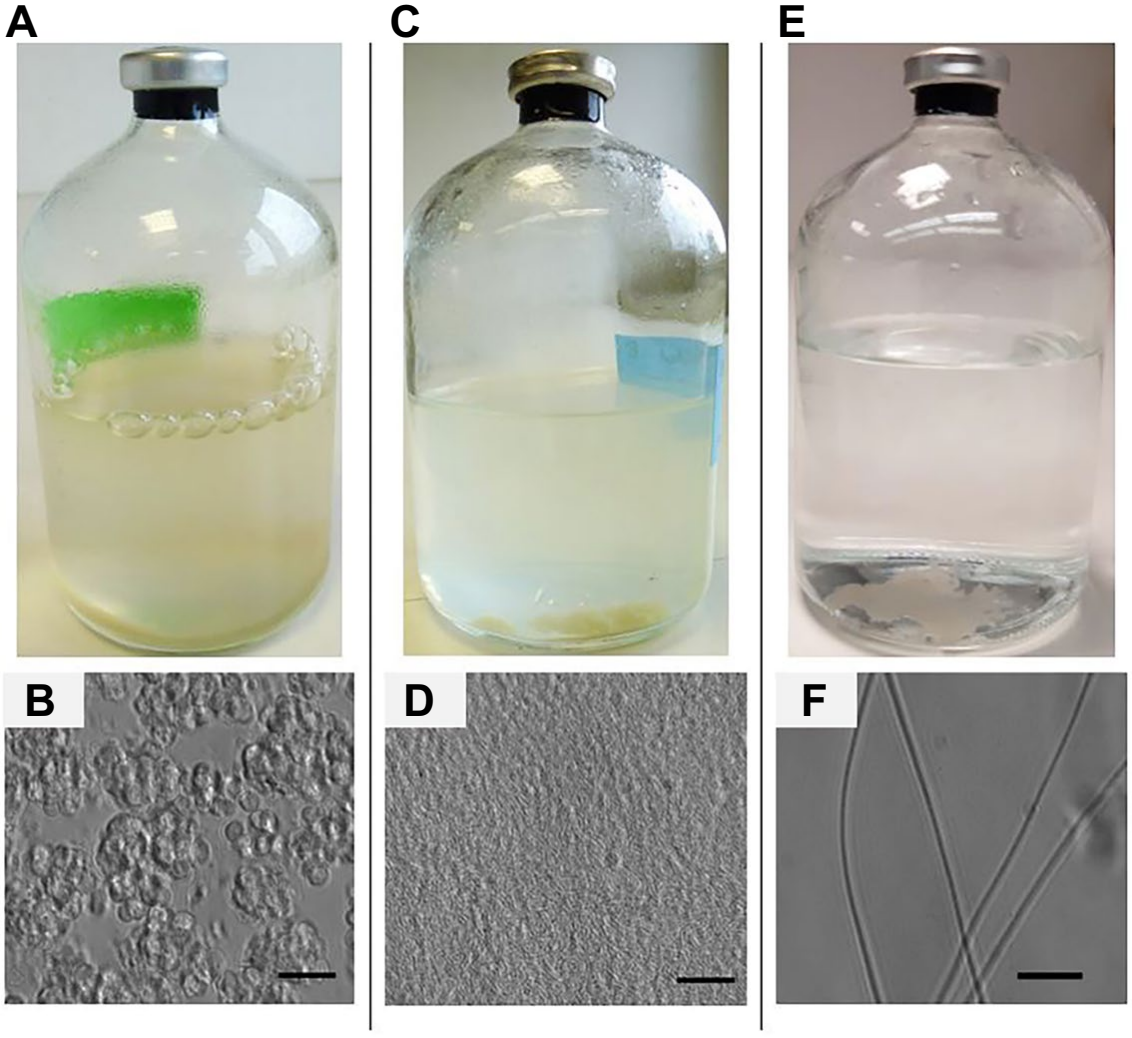
Photographs (week 3, top) and phase-contrast photomicrographs (week 7, bottom) of: **(A,B)** *Methanosarcina barkeri*, **(C,D)** *Methanobacterium formicicum*, and **(E,F)** *Methanothrix soehngenii*, grown in the BFS01 medium, bars indicate 10 μm.

It was observed that *M. barkeri* cells aggregate and settle at the bottom of the serum bottles (Figure 1A). This was confirmed by phase-contrast microscopy, which shows that the cells aggregate in irregular-sized cell clumps connected to each other (Figure 1B). In contrast, *M. formicicum* cells aggregate as small lumps which float in the medium under moderate agitation (100 rpm) or settle as non-motile agglomerates (Figure 1C). Phase contrast microscopy indicates that *M. formicicum* forms a cell colony resembling a sponge-like structure (Figure 1D). Finally, the growth of *M. soehngenii* appears as non-motile, showing very long and flexible filaments that tend to clump into characteristic bundles and settle at the bottom of the serum bottle (Figure 1E). The phase contrast microscopy picture of *M. soehngenii* cells shows a rod-like filament morphology (Figure 1F).

### 3.2. Substrate consumption, *COD* removal, product formation, and OD_600_

**Figure 2:**
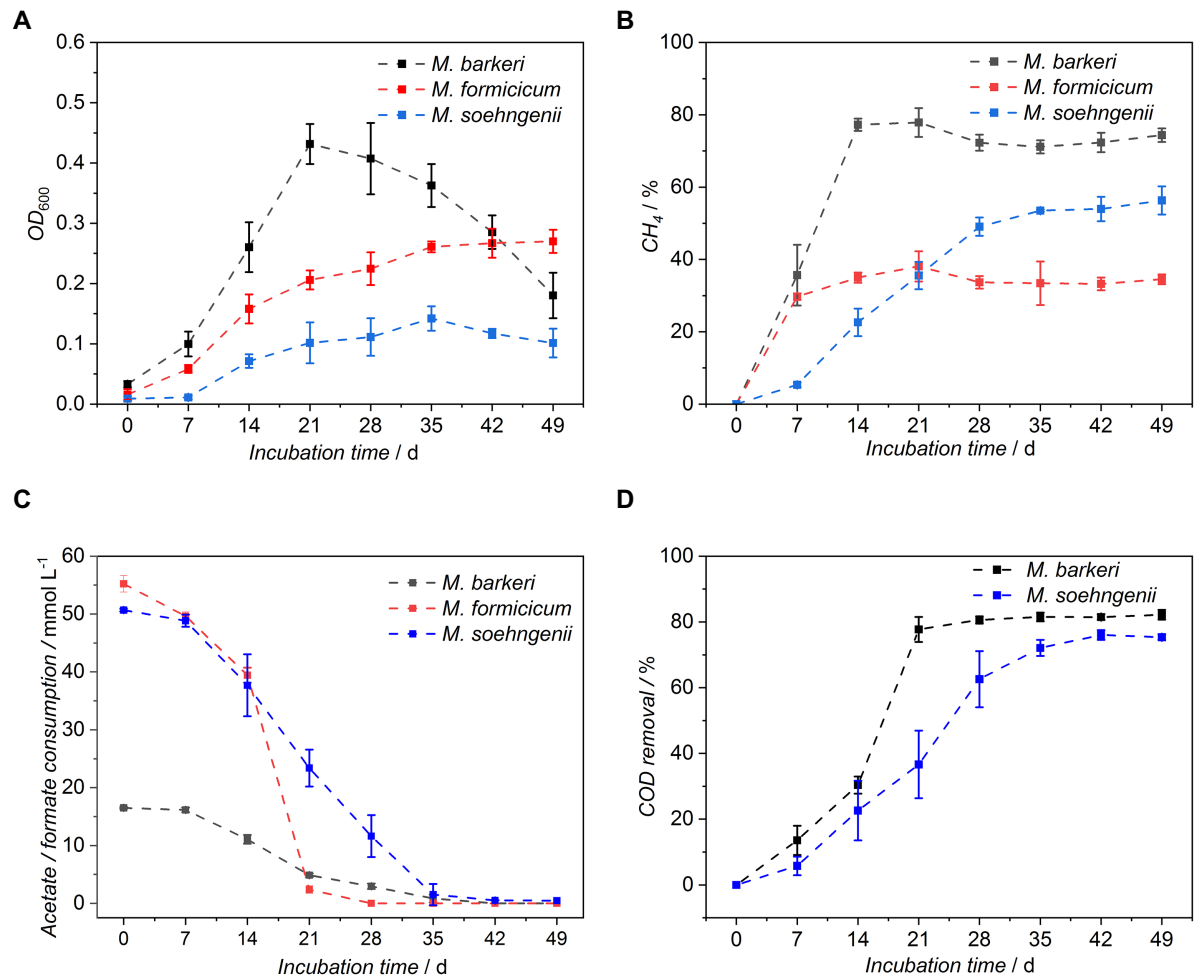
Growth characteristics of *Methanosarcina barkeri*, *Methanobacterium formicicum*, and *Methanothrix soehngenii* in the BFS01 medium: **(A)** OD_600_, **(B)** CH_4_ concentration in the headspace of the cultures, **(C)** acetate (blue and black dots) and formate (red dots) consumption, **(D)** *COD* removal, error bars indicate CI, *n* = 3.

To monitor the growth of *M. barkeri*, *M. formicicum*, and *M. soehngenii* in the BFS01 medium, substrate consumption, COD removal, CH_4_ formation, and OD_600_ were measured weekly for a period of 7 weeks (49 days). The optical density (OD_600_) in *M. barkeri* cultures increased and reached a maximum of 0.43 ± 0.03 at day 21, before it gradually decreased until day 49 (Figure 2A). Compared to *M. barkeri*, *M. formicicum* grew slower and progressively with a maximum OD_600_ of 0.27 ± 0.02 measured at day 49 (Figure 2A). In contrast, *M. soehngenii* displayed a lag phase of at least 7 days and a slower growth compared to *M. formicicum*, reaching its maximum OD_600_ of 0.14 ± 0.02 at day 35, after which it decreased until day 49.

The maximum headspace CH_4_ concentration for *M. barkeri* was measured at day 14 and remained nearly constant until day 21 (Figure 2B). From day 28 onwards, the CH_4_ concentration decreased slightly, but remained at an almost constant level until day 49. The acetate concentration during the initial 14 days (Figure 2C) only decreased by ~33% when the maximum CH_4_ concentration was reached. From day 7 onwards, acetate was consumed significantly each week in *M. barkeri* cultures as indicated by non-overlapping CI and was almost depleted by day 35 (c_day35_ = 0.84 ± 0.69 mmol L^−1^; Figure 2C).

*M. formicicum* already reached the plateau phase in terms of CH_4_ production at day 7 (Figure 2B), while the formate concentration in the serum bottles only decreased by ~10% (Figure 2C). Compared to the initial formate concentration in the cultures (i.e., 55.22 ± 1.42 mmol L^−1^), ~19% and ~67% of the formate was consumed during the second and third week after incubation, respectively. From day 28 onwards, formate was almost completely depleted in the *M. formicicum* cultures (c_day28_ = 2.37 ± 0.65 mmol L^−1^; Figure 2C) and from this stage on, the average CH_4_ concentration in the headspaces remained nearly constant until day 49 (Figure 2B).

In contrast to *M. barkeri* and *M. formicicum, M. soehngenii* reached a stationary phase of CH_4_ production after roughly 28 days with 49.1 ± 2.5% CH_4_ in the headspace (Figure 2B). The CH_4_ concentration increased further, albeit not significantly until day 49 to 56.3% ± 3.9%. It was observed that *M. soehngenii* gradually consumed acetate, which was nearly exhausted by day 35 (c_day35_ = 0.87 ± 1.54 mmol L^−1^; Figure 2C). The trend of acetate consumption in *M. soehngenii* cultures was nearly inversely proportional to the increase in CH_4_ concentration (Figure 2B).

The COD removal reached the plateau phase by ~day 21 and ~ day 28–35, for *M. barkeri* and *M. soehngenii,* respectively (Figure 2D). *M. formicicum* cultures were not analyzed for COD removal due to mixed provision of H_2_:CO_2_ (every 3–4 days) and formate. Furthermore, it is not possible to determine ΔCOD of formate fed cultures using COD kits, as it decomposes into CO and H_2_O on contact with sulfuric acid contained therein. However, our results indicate that the CH_4_ concentration alone is a sufficient indicator to monitor the growth of methanogens in all examined cultures using the BFS01 medium.

### 3.3. Physiological parameters: Specific growth rate ( μ ) and doubling time ( $t_{d}$ )

To estimate μ and $t_{d}$ of *M. barkeri, M. formicicum,* and *M. soehngenii* grown in the BFS01 medium, calculations based on either OD_600_ or CH_4_ concentration in the culture headspaces were performed and compared (for details please see SI: Calculations).

First, μ and $t_{d}$ of the three methanogens were estimated using OD_600_ measured during the growth period. The log-scale of the OD_600_ of *M. barkeri, M. formicicum*, and *M. soehngenii* cultures show that the growth was exponential between days 0 to 14, 0 to 14, and 7 to 14, respectively (Supplementary Figures S2A, S3A, S4A). The specific growth rates were similar for *M. barkeri* and *M. formicicum*, whereas the specific growth rate of *M. soehngenii* was ~2 times higher (Table 4; for more information about the curve fitting, see Supplementary Figures S2B, S3B, S4B).

**Table 4:** Specific growth rate (*μ*) and doubling time (*t_d_*) of *Methanosarcina barkeri, Methanobacterium formicicum* and *Methanothrix soehngenii* as functions of OD_600_ and CH_4_ concentration in the headspace of the cultures.

Parameter considered	OD_600_		% CH_4_		Literature values	
	*µ*/day^−1^	*t_d_*/day	*µ*/day^−1^	*t_d_*/day	*µ*/day^−1^	*t_d_*/day
*M. barkeri*	0.15 ± 0.01	4.73 ± 0.38	0.54 ± 0.04	1.29 ± 0.09	0.35–1.39 (Jetten et al., 1992)	0.5–2 (Jetten et al., 1992)
						1–2 (Patel et al., 2017)
*M. formicicum*	0.16 ± 0.01	4.26 ± 0.28	0.51 ± 0.01	1.37 ± 0.02	1.5 (Neil and James, 1980)	0.45 (Neil and James, 1980)
						0.6 (Neil et al., 1982)
*M. soehngenii*	0.27 ± 0.03	2.59 ± 0.25	0.21 ± 0.03	3.41 ± 0.53	0.1 (Jetten et al., 1992) 0.21 (Huser et al., 1982)	3.4 (Huser et al., 1982)
						3.24 (Touzel et al., 1988)

Errors indicate confidence interval (CI).

Subsequently, μ and $t_{d}$ of the three methanogens were estimated using CH_4_ accumulation in the headspaces. The CH_4_ concentration in the headspace at log-scale of *M. barkeri, M. formicicum*, and *M. soehngenii* cultures show that the growth was exponential between days 0 to 7, 0 to 7, and 7 to 14, respectively (Supplementary Figures S2C, S3C, S4C). The specific growth rates and corresponding doubling times were similar for *M. barkeri* and *M. formicicum*, whereas the doubling time of *M. soehngenii* was 2–3 times higher (Table 4; for more information about the curve fitting, see Supplementary Figures S2D, S3D, S4D).

### 3.4. Molecular biology

The purity of each methanogen grown in the BFS01 medium was confirmed by 16S rRNA gene-based amplicon sequencing and whole genome nanopore sequencing. The 16S rRNA gene-based amplicon sequencing analysis at the genus level indicated a relative abundance of 98.22% *Methanosarcina* spp., 99.98% *Methanobacterium* spp., and 99.97% *Methanothrix* spp. for *M. barkeri*, *M. formicicum,* and *M. soehngenii* cultures, respectively (data not shown). However, as the amplicon sequencing only covers the V3–V4 region, which does not allow for good differentiation down to the species level, but is limited to the genus or in some cases only the family level, whole genome nanopore sequencing was performed to verify species identity. Sequence assemblies built from the 16S rRNA gene amplicon reads showed high sequence similarity to entries in the 16S rRNA gene database (Table 5). Comparing the draft assemblies with all publicly available high-quality reference genomes for the three methanogens (Supplementary Tables S3–S5) indicates an average nucleotide identity (ANI) of 99.04% for *M. barkeri*, 96.55% for *M. formicicum* and 99.97% for *M. soehngenii* against their best hits in the NCBI reference databases (Table 5). The total length of the *M. formicicum* draft assembly is only 1.75 Mbp, which is only around 70% of the size of its best hit in the NCBI genbank database (accession: LN515531). This is a result of the very low sequencing depth for this sample, where the average read-mapping coverage is only around 5x, even when data down to Q7 (80% single base accuracy) compared to Q10 (90% single base accuracy) was used for the two other assembly. The *M. formicicum* draft assembly will also have a higher number of base positions that have not been error-corrected due to the very low sequencing coverage. This is also seen by the alignment percentage only being 83.16% for this assembly compared to 94.59% and 99.47% for the *M. barkeri* and *M. soehngenii* draft assemblies showing coverages of 8x and 18x, respectively. Furthermore, the *M. formicicum* draft assembly will have lower numbers of uncorrected bases both from the higher sequencing depth and the use of Q10 data.

**Table 5:** Whole genome assembly statistics (number of contigs, N50, GC-content, and average coverage), comparison to reference genomes [accession, average nucleotide identity (ANI), and alignment percentage (AP)], and best hit in the 16S rRNA gene reference database (accession, matching region in the reference sequence).

Strain origin	Species	Contigs	Assembly length	N50^§^	GC^#^ content (%)	Average coverage	Reference genome accession	ANI	AP	16S rRNA gene accession	region (bp)
DSM 800	*Methanosarcina barkeri*	70	4,417,454	106,578	39.04	8	NZ_CP009528	99.04	94.59	NR_118371	268–644
DSM 1535	*Methanobacterium formicicum*	48	1748095^*^	48,183	40.88	5	LN515531	96.55	83.16	NR_115168	68–650
DSM 3671	*Methanothrix soehngenii*	2	3,027,429	3,014,835	50.97	18	NC_015416	99.97	99.47	NR_102903	314–737

§ N50, shortest contig length to be included for covering 50% of the genome.

# GC-content, guanine-cytosine content.

* The total length of this assembly is much smaller than that of the reference genome.

## 4. Discussion

### 4.1. Good growth of the three methanogens in the BFS01 medium but the morphology of *Methanobacterium formicicum* changed

Culturing methanogens for potential application requires simple, general and user-friendly media. We have shown that *M. barkeri*, *M. formicicum,* and *M. soehngenii* can be efficiently cultivated in the BFS01 medium with low salt concentration and omission of resazurin, yeast extract and casitone. Members of the *Methanosarcinaceae* are reported to appear as nonmotile, irregular shaped spheroids bodies, occurring as packages of several cells or large aggregates (Jetten et al., 1992). This is in good agreement with the microscope image (Figure 1B), which showed typical irregular-sized cell clumps that are known from *M. barkeri* cultivation in other media (Zeikus, 1977; Bryant and Boone, 1987a; Jetten et al., 1992). Members of the *Methanobacteriaceae* are reported to have a rod- or coccoid-like cell shape (Bryant and Boone, 1987b; Dworkin et al., 2006; Demirel and Scherer, 2008). This contrasts with *M. formicicum* grown in the BFS01 medium, which resembled a sponge-like structure (Figure 1D). A different cell morphology has been reported for the same strain after 20 days of growth in a nearly similar medium to the DSM-120 medium, occurring as crooked rods (Battumur et al., 2016). Members of the *Methanosaetaceae/Methanotrichaceae* are reported to be rod-shaped non-spore-forming cells, which combine in long filaments, and often form large aggregates in unshaken cultures (Huser et al., 1982; Jetten et al., 1992). This is in agreement with our observations of *M. soehngenii* grown in the BFS01 medium (Figure 1F), which appeared as rod-like filaments. Therefore, we conclude that the use of a low salt concentration and the omission of chemically complex organic components did not hamper the growth of the three methanogens in the BFS01 medium, however did affect the cell morphology of *M. formicicum*. To shed light on the observed change in *M. formicicum* morphology, we advocate a follow-up study varying the salt concentration in the BFS01 medium or successively removing chemically complex organics from the DSM-120 medium.

### 4.2. Assessment of OD_600_, substrate consumption, *COD* removal, and product formation, during growth of *Methanosarcina barkeri*, *Methanobacterium formicicum*, and *Methanothrix soehngenii* in the BFS01 medium

The growth of microorganisms in liquid culture media is commonly monitored by measuring the OD_600_ which for dispersed cells correlates with the number of cells in the culture (Eppendorf, 2015; Tsuchiya et al., 2018). However, many factors such as size and shape as well as inactive biomass and other precipitates can affect the results of OD_600_ measurements (Eppendorf, 2015). The measured OD_600_ during *M. barkeri* growth peaked at ~day 21 and then decreased until day 49 (Figure 2A). It is known that the more microbial cells are present in the solution, less light reaches the spectrophotometric detector and vice versa. Therefore, we assume that during the initial 21 days of growth, irregular clumps formed in *M. barkeri* cultures (Figure 1B), increased in size and later led to increased irregular light scattering. Furthermore, we assumed that the methanol in *M. barkeri* cultures was completely depleted by day 21, as indicated by the stabilization of the pressure in the culture headspace (data not shown). Depletion of the carbon and energy source may have led to an increase in inactive cells in the cultures certainly influencing the OD_600_ measurement.

In contrast to *M. barkeri*, *M. formicicum*, and *M. soehngenii* seemed to grow slower. To support continuous growth, *M. formicicum* was routinely supplied with H_2_:CO_2_, meaning that its cell numbers were likely to increase constantly until a certain cell density (i.e., stationary phase) was reached. *M. soehngenii* is reported to be slow-growing, even under optimal conditions (Touzel et al., 1988; Jetten et al., 1992). This could explain why the OD_600_ of *M. formicicum* and *M. soehngenii* gradually increased. However, it is difficult to explain why the OD_600_ of *M. formicicum* and *M. soehngenii* remained lower than that of *M. barkeri*, e.g., at day 21, as the OD_600_ measurement can be affected by the typical cell density of the cultures (not measured). *M. formicum* and *M. soehngenii* appeared as rods and filaments, respectively (see Figures 1D,F). However, cultures of *M. formicicum* appeared to be more homogeneous but also denser than those of *M. soehngenii*. This means that *M. formicicum* scattered light more than *M. soehngenii*, which due to its filamentous growth, may have allowed a high but erratic spectrum of light to be transmitted directly to the detector. In general, *M. formicum* and *M. soehngenii*, appeared to be less light scattering than *M. barkeri,* presumably due to their cell shape and agglomeration behavior and therefore, inhomogeneous turbidity of the cultures.

The maximum CH_4_ concentration for *M. barkeri* was reached around day 14, while acetate consumption during the initial 14 days only decreased by ~33% (Figures 2B,C), which is likely due to its low affinity for acetate but high affinity for methanol (Bryant and Boone, 1987a; Jetten et al., 1989, 1992). Furthermore, the rapid increase in pressure in the culture headspaces of *M. barkeri* (data not shown) and the formation of gas bubbles over the initial 14–21 days of cultivation (Figure 1A) are consistent with the stoichiometry of methanogenesis from methanol (see Equation 5 in Table 3; Lackner et al., 2018). This indicates that *M. barkeri* grown in the BFS01 medium preferentially consumed methanol during its initial growth phase (i.e., the first 14–21 days), and then relied solely on acetate consumption for maintenance.

H_2_ and formate were consumed throughout growth of *M. formicicum* when both were present in the culture media. Formate was almost completely depleted by ~day 28 (Figure 2C) and from that point onwards, we assume that CH_4_ production relied primarily on CO_2_ and H_2_ consumption (see Figure 2B). The insignificant variation of CH_4_ concentration in the headspaces of *M. formicicum* cultures after depletion of formate is consistent with previous work reporting slightly slower growth of *M. formicicum* using formate than when using H_2_:CO_2_ (Neil and James, 1980; Neil et al., 1982). This is best explained by the respective stoichiometry of anaerobic H_2_ oxidation (Equation 3 in Table 3) and formate oxidation (Equation 4 in Table 3) showing that, four mol of H_2_ and four mol of formate each are oxidized to one mol of CH_4_. Since the maximum energy available when oxidizing H_2_ ( $\Delta G^{o\prime }$ *=* −135.6 kJ mol^−1^) and the energy available when oxidizing formate ( $\Delta G^{o\prime }$ *=* −130.1 kJ mol^−1^) are nearly equal, we conclude that after total depletion of formate in *M. formicicum* cultures, the CH_4_ concentration in the headspaces should not be significantly affected due to the non-stoichiometric provision of H_2_ and CO_2_. CH_4_ concentration in the headspaces of *M. formicicum* cultures remained <40% vs. CO_2_ concentration > 60% (see Supplementary Figure S1B). *M. formicicum* cultures were routinely flushed (every 3–4 days) with a H_2_:CO_2_ (50:50, v/v). Based on the stoichiometry of hydrogenotrophic methanogenesis, only 25% of the CO_2_ overpressurized in the culture headspaces was used for CH_4_ formation. The remaining CO_2_ as well as the CO_2_ derived from formate oxidation, i.e., 3 mol of CO_2_ (see Equation 4 in Table 3) was accumulated in the headspace. This explains why CH_4_ concentration in the headspaces did not reach 100% throughout growth of *M. formicicum*, even after the formate was almost completely depleted.

*M. soehngenii* grown in the BFS01 medium gradually consumed acetate to produce CH_4_, as similarly observed for other growth media (Huser et al., 1982; Jetten et al., 1989, 1992; Figures 2B,C). CH_4_ concentration in the headspace of *M. soehngenii* cultures (e.g., at day 49) showed a value of 56.3% ± 3.9% which is in good agreement with the stoichiometry of acetoclastic methanogenesis (see Equation 2 in Table 3). *M. soehngenii* cultures were not vented during the growth period. This explains why the cumulative shares of carbon equivalents of CH_4_ and CO_2_ in the headspace of *M. soehngenii* cultures (see Supplementary Figure S1C) were less than 100%, primarily due to the addition of N_2_:CO_2_ (50:50, v/v) to the serum bottles after inoculation.

COD removal in *M. barkeri* cultures was faster than in *M. soehngenii* cultures (Figure 2D). Unlike *M. barkeri*, *M. soehngenii* is reported as slow-growing (Touzel et al., 1988; Jetten et al., 1992), hence the lower Δ COD . COD removals <100%, as observed even when no more substrate (i.e., acetate or methanol) was available in *M. barkeri* and *M. soehngenii* cultures, can be related to microbial biomass formation.

### 4.3. Methane accumulation is better used to estimate the specific growth rate ( μ ) and doubling time ( $t_{d}$ ) of methanogens

Members of the *Methanosarcinaceae* (e.g., *M. barkeri*) and *Methanobacteriaceae* (e.g., *M. formicicum*) are known to have a rapid μ and short $t_{d}$ in contrast to members of the *Methanosaetaceae* (e.g., *M. soehngenii*), which are slow-growing and double their cell number after several days (Neil and James, 1980; Neil et al., 1982; Touzel et al., 1988; Jetten et al., 1992). Calculations based on OD_600_ indicated a higher μ and lower $t_{d}$ of *M. soehngenii* compared to *M. barkeri* and *M. formicicum*, which is somehow counterintuitive. From the obtained results, we conclude that OD_600_ cannot be used effectively to measure the growth of the methanogens, especially when they form aggregates as *M. barkeri* or filaments as *M. soehngenii,* which is consistent with existing reports (Dworkin et al., 2006).

As an alternative to OD_600_, we estimated μ and $t_{d}$ of the three methanogens using CH_4_ accumulation in the culture headspaces. The μ of *M. barkeri* and *M. formicicum* were nearly identical during their logarithmic phases, and were ~2.57, respectively, ~2.43 fold higher than that of *M. soehngenii* (Table 4). Similar, $\;t_{d}$ based on CH_4_ concentration in the headspace indicated that *M. barkeri* and *M. formicicum* cells doubled approximately every 1.29 days and 1.37 days, respectively, while *M. soehngenii* cells doubled approximately every 3.4 days. These results are consistent with literature indicating a fast growth of *M. barkeri* and *M. formicicum* unlike *M. soehngenii* (Touzel et al., 1988).

Although our calculations were only approximate as CH_4_ concentration in the headspace of the cultures was only measured once a week and daily or hourly measurements would be required for an accurate estimation of μ , the results for *M. barkeri* and *M. soehngenii* appeared to be consistent with literature values (Neil and James, 1980; Jetten et al., 1992). In contrast, *M. formicicum* appeared to grow slower compared to the literature (Neil and James, 1980), which, as mentioned earlier, could be due to the low salt concentration and/or the omission of resazurin, yeast extract and casitone in the BFS01 medium.

Based on our results we conclude, that μ and $t_{d}$ of *M. barkeri* and *M. soehngenii* in the BFS01 medium can be estimated by measuring CH_4_ accumulation in the headspace instead of OD_600_. However, μ and $t_{d}$ of *M. formicicum* differed from literature data but still allow sufficient growth monitoring.

## Data availability statement

The raw sequencing reads have been released and can be found here: https://www.ebi.ac.uk/ena/browser/view/PRJEB53211. Sequence data from MiSeq amplicon sequencing and Nanopore sequencing have been added as Supplementary data files (FASTA files).

## Author contributions

DD, JK, and FH: conceptualization. DD: investigation, visualization, and writing—original draft preparation. DD, SS, TH, IA, and AW: molecular biology analysis. DD, JK, FH, TH: formal analysis. DD, JK, FH, and AW: funding acquisition. JK, FH, and AW: supervision. DD, JK, FH, SS, TH, IA, and AW: writing—review and editing. All authors contributed to the article and approved the submitted version.

## Funding

DD gratefully acknowledges funding by the PhD student program of the DAAD (German academic exchange service, 57381412) and the transnational access of the BRISK2 project, which has received funding from the European Union’s Horizon 2020’s research and innovation programme under grant agreement number 731101. This work was supported by the Helmholtz-Association within the Research Programme Renewable Energies.

## Conflict of interest

The authors declare that the research was conducted in the absence of any commercial or financial relationships that could be construed as a potential conflict of interest.

## Publisher’s note

All claims expressed in this article are solely those of the authors and do not necessarily represent those of their affiliated organizations, or those of the publisher, the editors and the reviewers. Any product that may be evaluated in this article, or claim that may be made by its manufacturer, is not guaranteed or endorsed by the publisher.

## Abbreviations

AD: Anaerobic digestion
ANI: Average nucleotide identity
AP: Alignment percentage
CI: Confidence interval
COD: Chemical oxygen demand
DIET: Direct interspecies electron transfer
EAB: Electroactive bacteria
GC: Gas chromatograph
GC: Content, guanine-cytosine content
HPLC: High performance liquid chromatography
MET: Microbial electrochemical technologies
MIET: Mediated interspecies electron transfer
N50: Shortest contig length to be included for covering 50% of the genome
OD: Optical density
*µ*: specific growth rate
*t_d_*: doubling time
n: number of replicates
