# Supplementary material for: A unified and simple medium for growing model methanogens
Source: Front Microbiol. 2023 Jan 10;13:1046260. doi: 10.3389/fmicb.2022.1046260 (PMC9871610; doi:10.3389/fmicb.2022.1046260)
Supplement: Supplementary file 6 [file Data_Sheet_6.pdf]

Supplementary Information

Table of Content

Supplementary data files 2

Calculations 3

Specific growth rate 3

Doubling time 3

References 10

Figure S1. Percentage of CH_4, CO_2 and H_2 in the headspace of the cultures during growth of: (A) *M. barkeri*, (B) *M. formicicum*, (C) *M. soehngenii* in the BFS01 medium, n = 3, error bars indicate confidence interval (CI) 4

Figure S2. Specific growth rate (μ) of M. barkeri in the BFS01 medium, based on OD_{600} and CH_4 concentration in the headspace of the cultures: (A) log-scaled of OD_{600} vs time (exponential baseline = 0.05), (B) Exponential curve fitting functions based on OD_{600}, (C) log-scaled of CH_4 % vs time (exponential baseline = 10), (D) Exponential curve fitting functions based on CH_4, n = 3 5

Figure S3. Specific growth rate (μ) of M. formicicum in the BFS01 medium, based on OD_{600} and CH_4 concentration in the headspace of the cultures: (A) log-scaled of OD_{600} vs time (exponential baseline = 0.05), (B) Exponential curve fitting functions based on OD_{600}, (C) log-scaled of CH_4 % vs time (exponential baseline = 10), (D) Exponential curve fitting functions based on CH_4, n = 3 5

Figure S4. Specific growth rate (μ) of M. soehngenii in the BFS01 medium, based on OD_{600} and CH_4 concentration in the headspace of the cultures: (A) log-scaled of OD_{600} vs time

(exponential baseline = 0.05), (B) Exponential curve fitting functions based on OD_{600}, (C) log-scaled of CH_4 % vs time (exponential baseline = 10), (D) Exponential curve fitting functions based on CH_4, n = 3 6

Table S1. recommended media used for preculturing the used methanogenic strains 7

Table S2. Composition of trace elements SL-10 and Vitamin solution-10 8

Table S3. Average nucleotide identity (ANI, upper in blue)/Alignment Percentage (AP, lower in black) comparison between genome assemblies of Methanosarcina barkeri. 9

Table S4. Average nucleotide identity (ANI, upper in blue)/Alignment Percentage (AP, lower in black) comparison between genome assemblies of Methanobacterium formicicum 9

Table S5. Average nucleotide identity (ANI, upper in blue)/Alignment Percentage (AP, lower in black) comparison between genome assemblies of Methanothrix soehngenii 9

Supplementary data files

Assembled 16S V3-V4 sequences from the amplicon-sequencing of DSM 800, DSM 1535, and DSM 3671 are included in the supplementary 16S.fasta file (Data Sheet 1)

The draft genome assemblies used for whole genome alignment studies are found in the following files:

- Data Sheet 2_*M. barkeri*_DSM 800_assembly_q10.fasta
- Data Sheet 3_*M. formicicum*_ DSM 1535_assembly_q10.fasta
- Data Sheet 4_*M. formicicum*_ DSM 1535_assembly_q7.fasta
- Data Sheet 5_*M. soehngenii*_ DSM 3671_assembly_q10.fasta

Calculations

Specific growth rate

The specific growth rate (μ) indicates the biomass increase over time with respect to the initial biomass concentration and is determined using the exponential phase of growth (or log phase) in which cells divide regularly by binary fission and grow by geometric progression. The exponential phase is easily distinguished by plotting the logarithm (Log_{10}) of the cell concentration and/or product formation versus time. The exponential phase is then fitted with a regression line to determine the growth function and the correlation factor R^2, using the exponential curve fitting function. The equation obtains is in the form:

$$x = x_0 \cdot e^{\mu \cdot t} \qquad \text{(eq. S1)}$$

with x_0 indicating the starting population, μ the specific growth rate, t the time (min, hrs, days) and x the population after t (min, hrs, days).

To calculate the growth rate of each methanogen, the logarithmic phase of growth was determined by considering the point preceding the exponential baseline automatically indicated in excel 2019 (i.e., the beginning of the acceleration phase) and the point preceding the stationary phase (i.e., the beginning of the decline phase).

Doubling time

The doubling time (t_d) indicates the time required for a population of microorganisms, depending on their type and the environmental conditions, to double in number by binary fission. t_d is calculated directly from the growth rate by rearranging equation S1 as follow:

$$x = x_0 \cdot e^{\mu \cdot t} \quad \Rightarrow \quad t = \frac{\ln(\frac{x}{x_0})}{\mu}$$

As the doubling time t_d is the time required for the population size x to increase from x_0 to $2x_0$, the equation is simplified to:

$$t_d = \frac{ln(\frac{2x_0}{x_0})}{\mu} = \frac{ln(2)}{\mu} \qquad \text{(eq. S2)}$$

This equation was used to calculate the doubling time of each methanogenic strain studied in this work.

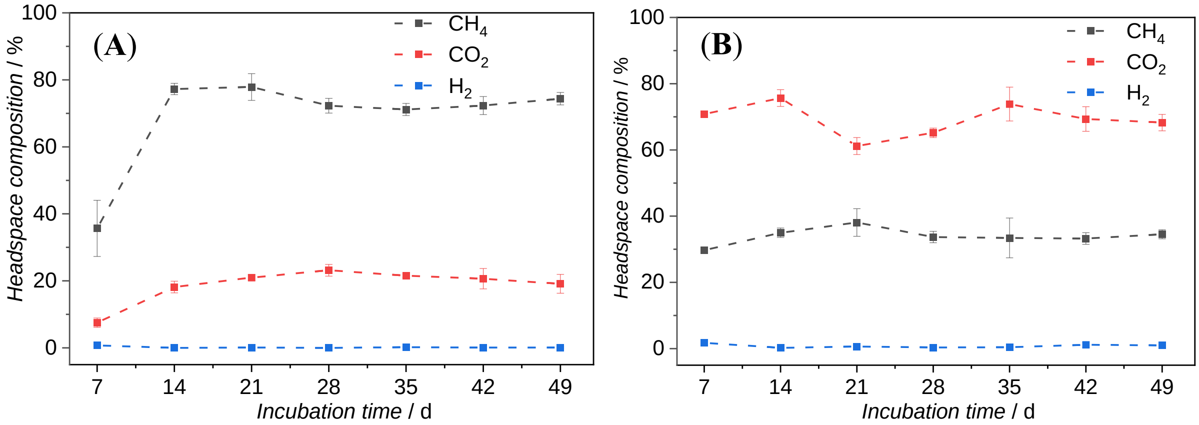

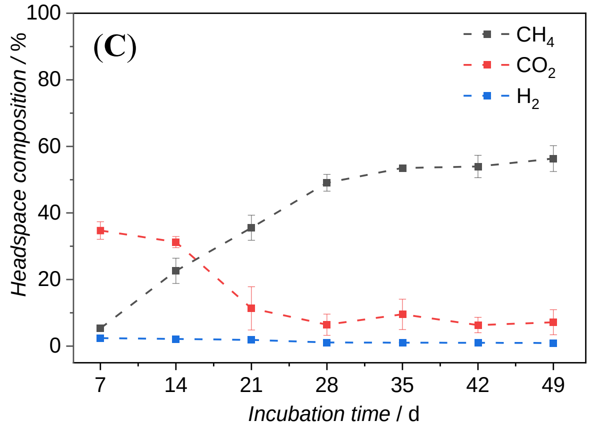

Figure S1. Percentage of CH_4, CO_2 and H_2 in the headspace of the cultures during growth of: (A) *M. barkeri*, (B) *M. formicicum*, (C) *M. soehngenii* in the BFS01 medium, n = 3, error bars indicate confidence interval (CI)

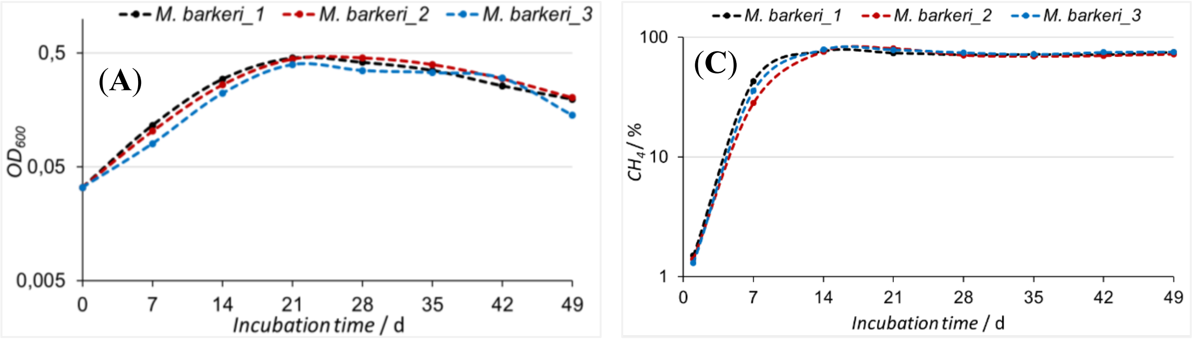

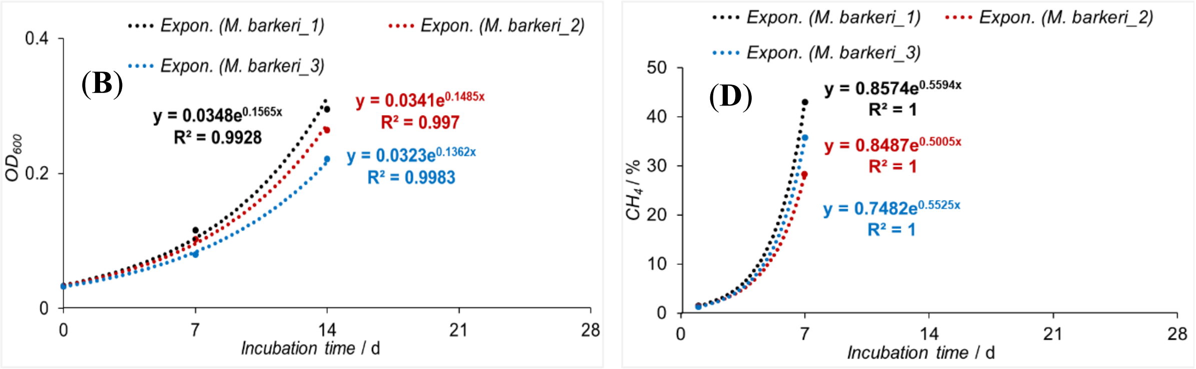

Figure S2. Specific growth rate (μ) of *M. barkeri* in the BFS01 medium, based on OD_{600} and CH_4 concentration in the headspace of the cultures: (A) log-scaled of OD_{600} vs time (exponential baseline = 0.05), (B) Exponential curve fitting functions based on OD_{600}, (C) log-scaled of CH_4 % vs time (exponential baseline = 10), (D) Exponential curve fitting functions based on CH_4, n = 3

Figure 2b still indicates a rapid CH_4 production by *M. barkeri* between days 7 and 14, while Figure S2c already indicates the start of the decline phase by ~day 7. Calculation based solely on data of day 7 and 14, indicated μ and t_d of 0.11±0.03 day^{-1} and 6.48±2.04 days, respectively (plot not shown). This slow-down of μ during the second incubation week confirms that the lag phase of *M. barkeri* occurred, as shown in Figure S2c, during the first 7 days of incubation.

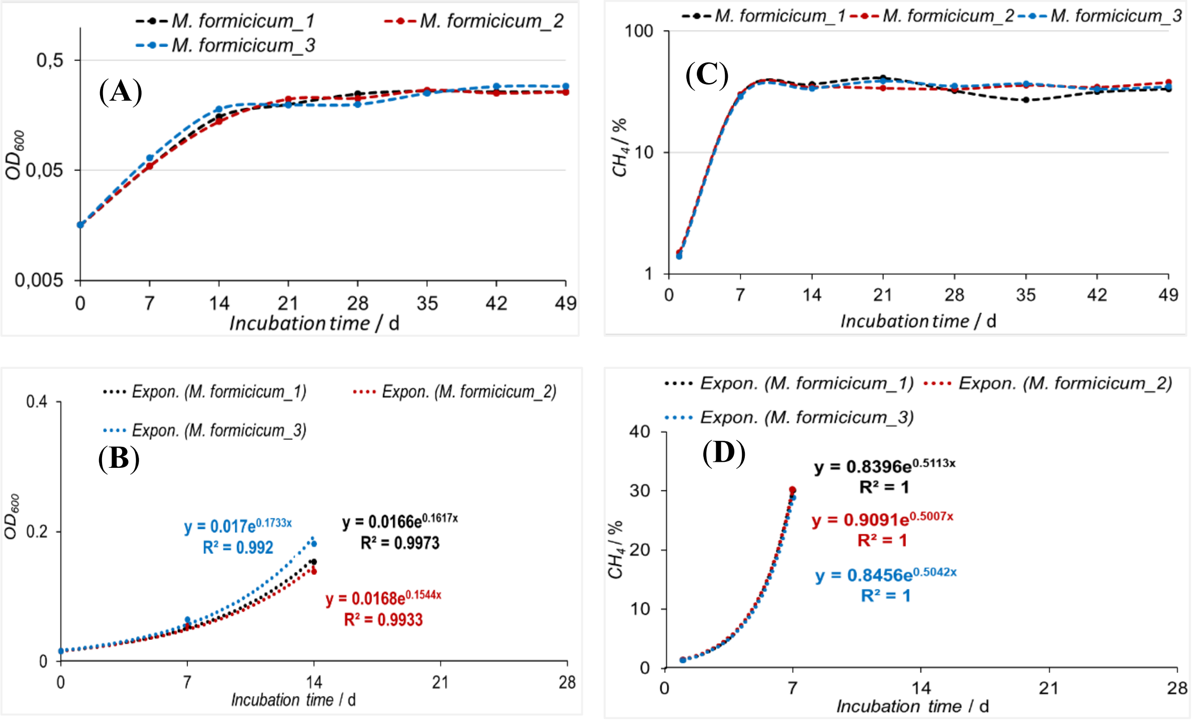

Figure S3. Specific growth rate (μ) of *M. formicicum* in the BFS01 medium, based on OD_{600} and CH_4 concentration in the headspace of the cultures: (A) log-scaled of OD_{600} vs time (exponential baseline = 0.05), (B) Exponential curve fitting functions based on OD_{600}, (C) log-

scaled of CH_4 % vs time (exponential baseline = 10), (D) Exponential curve fitting functions based on CH_4, n = 3

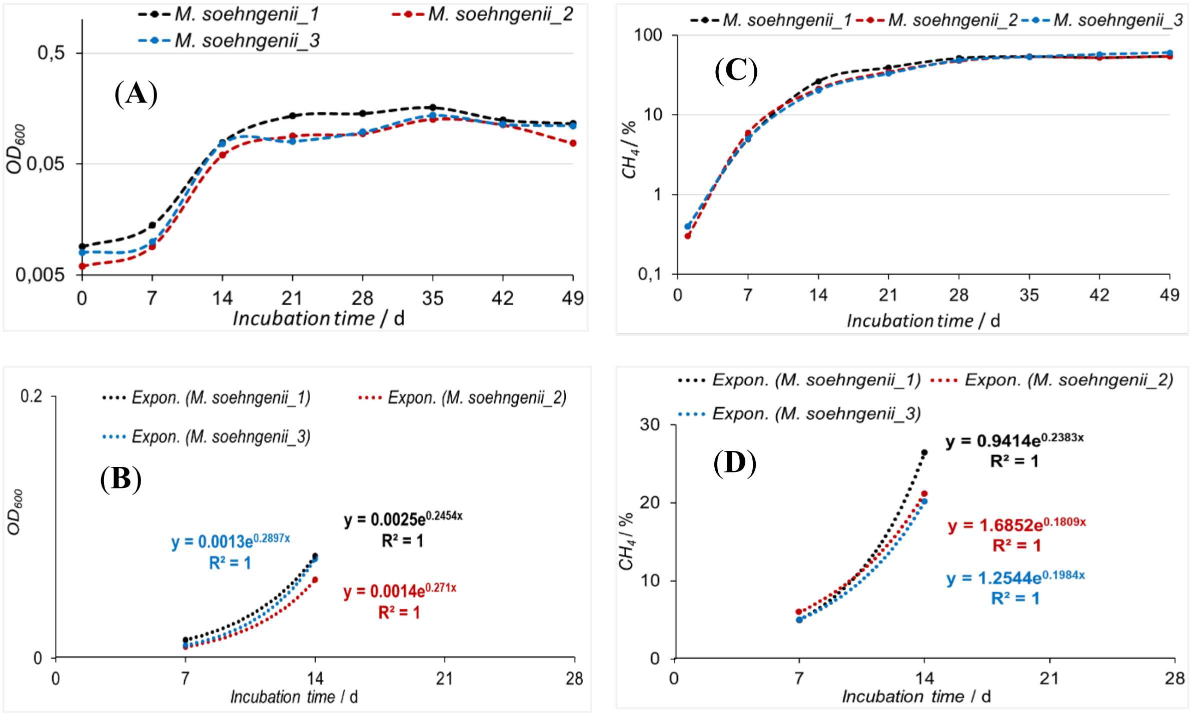

Figure S4. Specific growth rate (μ) of *M. soehngenii* in the BFS01 medium, based on OD_{600} and CH_4 concentration in the headspace of the cultures: (A) log-scaled of OD_{600} vs time (exponential baseline = 0.05), (B) Exponential curve fitting functions based on OD_{600}, (C) log-scaled of CH_4 % vs time (exponential baseline = 10), (D) Exponential curve fitting functions based on CH_4, n = 3

Table S1. Recommended media used for preculturing the used methanogenic strains

Component	Amount	Unit
M. barkeri (DSM-120 medium)		
K_2HPO_4	0.35	g L^{-1}
KH_2PO_4	0.23	g L^{-1}
NH_4Cl	0.50	g L^{-1}
$MgSO_4$ x 7 H_2O	0.50	g L^{-1}
$CaCl_2$ x 2 H_2O	0.25	g L^{-1}
NaCl	2.25	g L^{-1}
$FeSO_4$ x 7 H_2O solution (0.1% w/v in 0.1 N H_2SO_4)	2.00	mL L^{-1}
Trace element solution SL-10	1.00	mL L^{-1}
Yeast extract (OXOID)	2.00	g L^{-1}
Casitone (BD BBL)	2.00	g L^{-1}
Na-resazurin solution (0.1% w/v)	0.50	mL L^{-1}
$NaHCO_3$	2.00	g L^{-1}
Wolin's vitamin solution-10	1.00	mL L^{-1}
Methanol	10.00	mL L^{-1}
L-Cysteine-HCl x H_2O	0.30	g L^{-1}
Na_2S x 9 H_2O	0.30	g L^{-1}
Distilled water	1000.00	mL L^{-1}
M. formicicum (DSM-141 medium)		
KCl	0.34	g L^{-1}
$MgCl_2$ x 6 H_2O	4.00	g L^{-1}
NH_4Cl	0.25	g L^{-1}
$MgSO_4$ x 7 H_2O	3.45	g L^{-1}
$CaCl_2$ x 2 H_2O	0.14	g L^{-1}
K_2HPO_4	0.14	g L^{-1}
NaCl	18.00	g L^{-1}
$Fe(NH_4)_2(SO_4)_2$ x 6 H_2O solution (0.1% w/v)	2.00	mL L^{-1}
Trace element solution SL-10	1.00	mL L^{-1}
Yeast extract (OXOID)	2.00	g L^{-1}
Trypticase peptone (BD BBL)	2.00	g L^{-1}
Na-resazurin solution (0.1% w/v)	0.50	mL L^{-1}
$NaHCO_3$	5.00	g L^{-1}
Wolin's vitamin solution-10	1.00	mL L^{-1}
L-Cysteine-HCl x H_2O	0.50	g L^{-1}
Na_2S x 9 H_2O	0.50	g L^{-1}
Distilled water	1000.00	mL L^{-1}
M. soehngenii (CP Anaerobic medium) (Stams et al. 1993)		
KH_2PO_4	0.41	g L^{-1}
$Na_2HPO_4.2H_2O$	0.53	g L^{-1}
NH_4Cl	0.30	g L^{-1}
$MgCl_2$ x 6 H_2O	4.00	g L^{-1}
$CaCl_2$ x 2 H_2O	0.11	g L^{-1}
NaCl	0.30	g L^{-1}
Trace element solution SL-10	1.00	mL L^{-1}
Na-acetate	5.44	g L^{-1}
Na-resazurin solution (0.1 % w/v)	0.50	mL L^{-1}
$NaHCO_3$	4.00	g L^{-1}
Wolin's vitamin solution-10	1.00	mL L^{-1}
L-Cysteine-HCl x H_2O	0.50	g L^{-1}
Na_2S x 9 H_2O	0.24	g L^{-1}
Distilled water	1000.00	mL L^{-1}

Table S2. Composition of trace elements SL-10 and Vitamin solution-10

Component	Amount	Unit
**Trace elements SL-10**		
$FeCl_2 \times 4 H_2O$	1500	mg L^{-1}
HCl	10	mL L^{-1}
$ZnCl_2$	70	mg L^{-1}
$MnCl_2 \times 4 H_2O$	100	mg L^{-1}
H_3BO_3	6	mg L^{-1}
$CoCl_2 \times 6 H_2O$	190	mg L^{-1}
$CuCl_2 \times 2 H_2O$	2	mg L^{-1}
$NiCl_2 \times 6 H_2O$	24	mg L^{-1}
$Na_2MoO_4 \times 2 H_2O$	36	mg L^{-1}
**Vitamin solution**		
Biotin	2	mg L^{-1}
Folic acid	2	mg L^{-1}
Pyridoxine	10	mg L^{-1}
Thiamine	5	mg L^{-1}
Riboflavin	5	mg L^{-1}
Nicotinic acid	5	mg L^{-1}
Calcium pantothenate	5	mg L^{-1}
Vitamin B12	0.1	mg L^{-1}
p-Aminobenzoate	5	mg L^{-1}
Lipoic acid	5	mg L^{-1}

Table S3. Average nucleotide identity (ANI, upper in blue)/Alignment Percentage (AP, lower in black) comparison between genome assemblies of *Methanosarcina barkeri*.

	NZ_CP009526	NZ_CP009517	DSM 800_assembly	NZ_CP008746	NZ_CP009528	NZ_CP009530
NZ_CP009526		86.48	90.18	91.07	91.06	91.1
NZ_CP009517	57.87		85.56	86.4	86.34	86.26
DSM 800_assembly	67.59	54.86		98.5	**99.04**	98.56
NZ_CP008746	71	59.17	87.36		99.49	99.44
NZ_CP009528	71.05	58.7	**94.59**	92.1		99.54
NZ_CP009530	71.53	59.02	88.39	92.31	92.98	

Table S4. Average nucleotide identity (ANI, upper in blue)/Alignment Percentage (AP, lower in black) comparison between genome assemblies of *Methanobacterium formicicum*

	NZ_LN734822	LN515531	NZ_CP006933	BBES01	DSM 1535_assembly_q10	DSM 1535_assembly_q7
NZ_LN734822		98.66	99.55	98.5	91.82	95.2
LN515531	85.85		98.74	99.85	95.55	**96.55**
NZ_CP006933	93.48	86.66		98.6	92.88	95.24
BBES01	85.97	98.69	86.67		95.3	**96.43**
DSM 1535_assembly_q10	26.28	49.16	28.66	47.99		96.45
DSM 1535_assembly_q7	70.62	83.16	71.06	**82.8**	50.67	

Table S5. Average nucleotide identity (ANI, upper in blue)/Alignment Percentage (AP, lower in black) comparison between genome assemblies of *Methanothrix soehngenii*

	NC_015416	DSM 3671_assembly_q10
NC_015416		**99.97**
DSM 3671_assembly_q10	**99.47**	

References

Stams AJM, Dijk JBV, Dijkema C, Plugge CM (1993) Growth of Syntrophic Propionate-Oxidizing Bacteria with Fumarate in the Absence of Methanogenic Bacteria. Appl. Environ. Microbiol. 59:1114–1119. https://doi.org/10.1128/aem.59.4.1114-1119.1993
